# A Novel κ-Carrageenase from Marine Bacterium *Rhodopirellula sallentina* SM41: Heterologous Expression, Biochemical Characterization and Salt-Tolerance Mechanism Investigation

**DOI:** 10.3390/md20120783

**Published:** 2022-12-16

**Authors:** Yong-Hui Zhang, Yi-Ying Chen, Xiao-Yan Zhuang, Qiong Xiao, Jun Chen, Fu-Quan Chen, Qiu-Ming Yang, Hui-Fen Weng, Bai-Shan Fang, An-Feng Xiao

**Affiliations:** 1 College of Food and Biological Engineering, Jimei University, Xiamen 361021, China; 2 Fujian Provincial Engineering Technology Research Center of Marine Functional Food, Xiamen 361021, China; 3 Xiamen Key Laboratory of Marine Functional Food, Xiamen 361021, China; 4 College of Chemistry and Chemical Engineering, Xiamen University, Xiamen 361021, China

**Keywords:** κ-carrageenase, κ-carrageenan, heterologous expression, salt-tolerance

## Abstract

κ-carrageenases are members of the glycoside hydrolase family 16 (GH16) that hydrolyze sulfated galactans in red algae, known as κ-carrageenans. In this study, a novel κ-carrageenase gene from the marine bacterium *Rhodopirellula sallentina* SM41 (RsCgk) was discovered via the genome mining approach. There are currently no reports on κ-carrageenase from the *Rhodopirellula* genus, and RsCgk shares a low identity (less than 65%) with κ- carrageenase from other genera. The RsCgk was heterologously overexpressed in *Escherichia coli* BL21 and characterized for its enzymatic properties. RsCgk exhibited maximum activity at pH 7.0 and 40 °C, and 50% of its initial activity was retained after incubating at 30 °C for 2 h. More than 70% of its activity was maintained after incubation at pH 6.0–8.0 and 4 °C for 24 h. As a marine derived enzyme, RsCgk showed excellent salt tolerance, retaining full activity in 1.2 M NaCl, and the addition of NaCl greatly enhanced its thermal stability. Mass spectrometry analysis of the RsCgk hydrolysis products revealed that the enzyme had high degradation specificity and mainly produced κ-carrageenan disaccharide. Comparative molecular dynamics simulations revealed that the conformational changes of tunnel-forming loops under salt environments may cause the deactivation or stabilization of RsCgk. Our results demonstrated that RsCgk could be utilized as a potential tool enzyme for efficient production of κ-carrageenan oligosaccharides under high salt conditions.

## 1. Introduction

Carrageenan is a water-soluble sulfated linear polysaccharide that is generally extracted from red algae [1]. Carrageenan polysaccharide is composed of 3-linked β-D-ga- lactopyranose (G-units) and 4-linked α-D-galactopyranose (D-units) or 4-linked 3,6-anhydro-α-D-galactopyranose (DA-units). Based on the condition of sulphate esters (S) and DA units, carrageenans are classified into κ-carrageenan, ι-carrageenan, and λ-carrageenan [2]. Carrageenan oligosaccharide is generally recognized as safe (GRAS) by the US Food and Drug Administration [3]. Due to the widespread cultivation of red algae, carrageenan has been extensively studied for its extraction and degradation strategies, and hydrolyzed carrageenan oligosaccharide has been widely used in food industries as an additive for antioxidant properties, gelling, or thickening [4], as well as in the cosmetic and pharmaceutical industries as emulsifiers and carriers [5]. Recent studies have shown that carrageenan oligosaccharide exhibits excellent biological activity, including antioxidant, anti-tumor, and anti-inflammatory activity [6,7]. 

κ-carrageenases (EC 3.2.1.83) can catalyze the degradation of carrageenan polysaccharides through specific cleavage of the β-1,4 linkages in κ-carrageenan in an endolytic manner and produce a range of κ-carrageenan oligosaccharides. Thus, the development of κ-carrageenases is vital for the efficient production of carrageenan oligosaccharides. For instance, carrageenan oligosaccharides produced by κ-carrageenase Car3206 from an Antarctic strain of *Polaribacter* sp. NJDZ03 [8] showed antioxidant capacity toward hydroxyl radicals and DPPH radicals. Li et al. found that a κ-carrageenase CgkA from marine bacterium *Vibrio* sp. SY01 mainly produced κ-carradiaose, which exhibited antioxidant activity [9]. In recent years, researchers have identified various enzymes from marine organisms that have great potential for extensive biological applications [10,11,12,13,14]. κ-carrageenases are largely derived from marine organisms, including animals, bacteria, algae, and fungi. Due to the high-salt conditions in which these organisms live and evolve, considerable salt-related properties, including salt activation/dependence and salt tolerance, are often found in marine-derived κ-carrageenases. Cui et al. reported that the κ-carrageenase R-Cly-κ-CAR from the marine bacterium *Cellulophaga lytica* strain N5-2 required 0.6% NaCl to obtain the highest enzymatic activity [15]. The activity of κ-carrageenase CgkA from marine bacterium *Pedobacter hainanensis* NJ-02 was enhanced nearly two-fold with the addition of 300 mM NaCl [16]. κ-carrageenase from *Pseudoalteromonas carrageenovora* ASY5 exhibited its highest activity at 1400 mM NaCl [17].

Extraction of carrageenan by sodium and potassium salts is a commonly used industrial method to extract carrageenan from red algae [18,19]. The high concentration of sodium and potassium salts used in the production process remains in the carrageenan extraction wastes, impeding the further utilization of these wastes. Therefore, it is of great practical significance to develop carrageenases that are resistant to high concentrations of sodium and potassium salts. Despite the extensive discovery of new κ-carrageenase tools for carrageenan oligosaccharide preparation and their commonly observed salt-related properties, the research into the development of κ-carrageenase with high salt tolerance, especially tolerance to high concentration potassium salts, is scarce. κ-carrageenase R-Cly-κ-CAR from the marine bacterium *Cellulophaga lytica* strain N5-2 only retained 23.6% of its activity in 100 mM KCl [15]. κ-carrageenase from *Pseudoalteromonas carrageenovora* ASY5 retained about 50% of its activity in 150 mM KCl, and fully deactivated in 200 mM KCl [17].

In this study, a novel κ-carrageenase from the marine bacterium *Rhodopirellula sallentina* SM41 (RsCgk) was discovered using the genome mining approach and expressed in *Escherichia coli* BL21. The enzymatic performance of RsCgk was characterized, especially for its salt-related properties. RsCgk exhibited high salt tolerance toward both sodium and potassium salt. The degradation products of κ-carrageenan were analyzed via ESI–MS. In addition, molecular dynamics analysis was performed to investigate the structural basis for the salt-tolerance properties of RsCgk.

## 2. Results and Discussion

### 2.1. RsCgk Sequence Information

**Figure 1:**
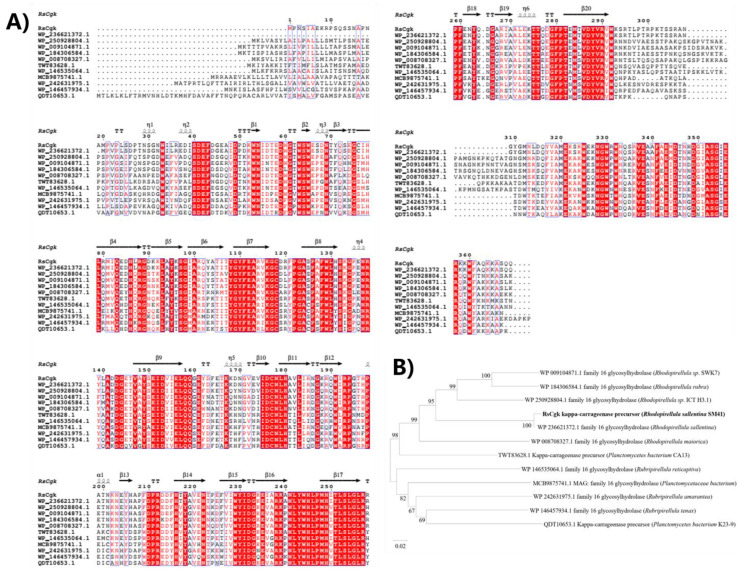
Multiple sequence alignment (**A**) and phylogenetic tree (**B**) of RsCgk with other κ-carrageenases deposited in NCBI database. The amino acid sequences of κ-carrageenases derived from *Rhodopirellula sallentina* (NCBI ID: WP_236621372.1), *Rhodopirellula* sp. ICT_H3.1 (NCBI ID: WP_250928804.1), *Rhodopirellula* sp. SWK7 (NCBI ID. WP_009104871.1), *Rhodopirellula rubra* (NCBI ID: WP_184306584.1), *Rhodopirellula maiorica* (NCBI ID: WP_008708327.1), *Planctomycetes bacterium* CA13 (NCBI ID TWT83628.1), *Rubripirellula reticaptiva* (NCBI ID: WP_146535064.1), *Planctomycetaceae bacterium* (NCBI ID: MCB9875741.1), *Rubripirellula amarantea* (NCBI ID: WP_242631975.1), *Rubripirellula tenax* (NCBI ID: WP_146457934.1), and *Planctomycetes bacterium* K23_9 (NCBI ID: QDT10653.1) were used for alignment and phylogenetic tree analysis.

The *Rhodopirellula* genus is a member of Planctomycetes, a ubiquitous bacterial phylum. They play a significant role in marine systems, especially in global carbon and nitrogen cycles [20,21]. Despite many κ-carrageenases being identified from marine bacteria such as *Pseudoalteromonas* [22], *Zobellia* [23], and *Cellulophaga* [24], there are still no reports on κ-carrageenases from the *Rhodopirellula* genus. To expand the toolbox of κ-carrageenase for industrial applications, we found a novel κ-carrageenase sequence from the marine bacterium *Rhodopirellula sallentina* SM41 (RsCgk, NCBI ID: EMI53032.1) using database mining. RsCgk contains 369 amino acids, with a calculated molecular weight of 42.5 kDa, and shares a low similarity with other κ-carrageenases deposited in the NCBI database (Figure 1A). The similarity of RsCgk with κ-carrageenases from different genera was less than 65%, indicating that it may have unique enzyme characteristics. A phylogenetic tree was built according to the sequence alignment (Figure 1B), and the results revealed that RsCgk is situated in the same clade as glycoside hydrolase family 16 (GH16).

### 2.2. Expression and Purification of RsCgk

**Figure 2:**
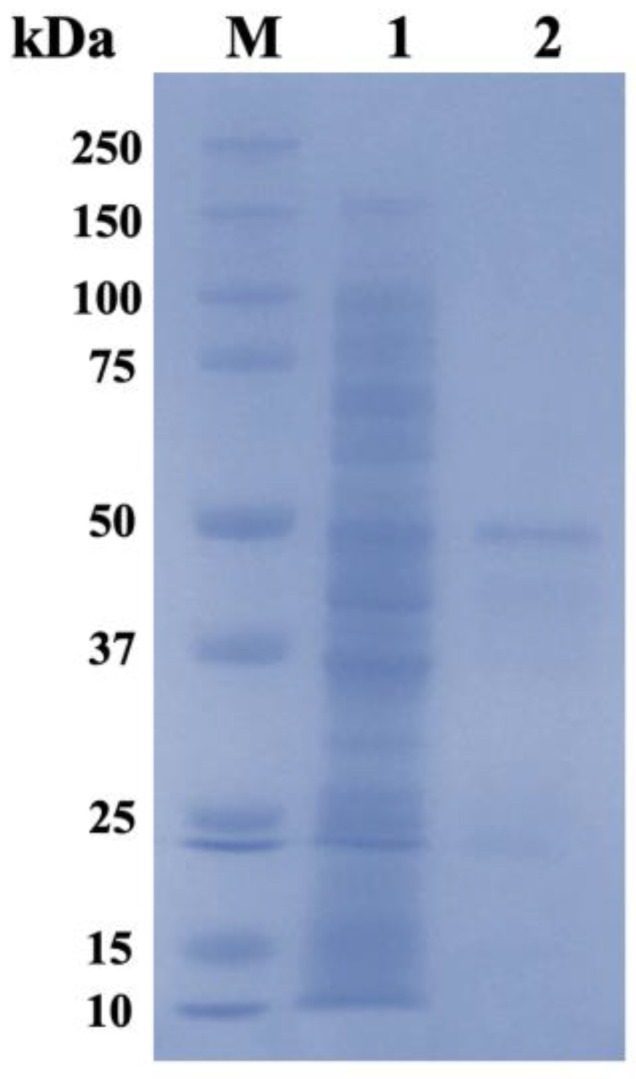
SDS-PAGE profiles of crude and purified RsCgk. M, protein marker; Lane 1, crude RsCgk; Lane 2, purified RsCgk.

The recombinant strain BL21 (DE3) that carried the pET-28a(+)-RsCgk plasmid was induced by IPTG to produce recombinant RsCgk, which was further purified by a Ni-NTA agarose column. The result of SDS-PAGE (Figure 2) indicated that the molecular mass of the expressed RsCgk was about 42.5 kDa, which was consistent with the theoretical molecular mass of the enzyme according to the amino acid sequence of RsCgk. The enzymatic activity of RsCgk was 32.9 U/mg. The purified RsCgk was used in further investigations of enzymatic characteristics.

### 2.3. Biochemical Characterization of RsCgk

**Figure 3:**
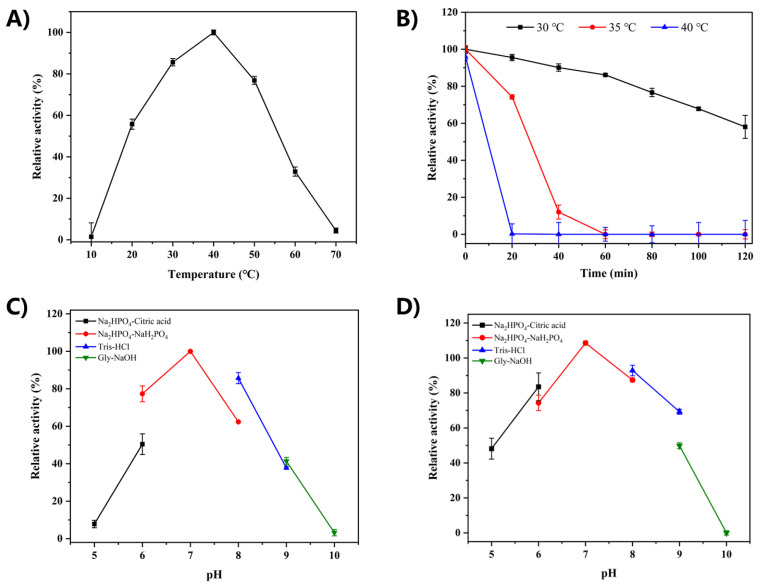
Effects of pH and temperature on the activity and stability of RsCgk. (**A**) The optimal temperature of RsCgk. The activities of RsCgk were assayed at various temperatures (10–70 °C). (**B**) The thermal stability of RsCgk. The residual activities of RsCgk were assayed after being kept at different temperatures (30 °C, 35 °C, and 40 °C) for 120 min. Relative activity is calculated as a percentage of initial activity. (**C**) The optimal pH of RsCgk. RsCgk activities were assayed in Na_2_HPO_4_–citric acid buffer (50 mM, pH 5.0–6.0), Na_2_HPO_4_–NaH_2_PO_4_ buffer (50 mM, pH 6.0–8.0), Tris–HCl buffer (50 mM, pH 8.0–9.0), and glycine–sodium hydroxide buffer (50 mM, pH 9.0–10.0). Relative RsCgk activities were calculated using the activity measured at optimum pH as 100%. (**D**) The pH stability of RsCgk. The residual activities of RsCgk were measured after the enzyme was incubated in the buffers described above (50 mM, pH 5.0–10.0) at 4 °C for 24 h. Residual RsCgk activities were calculated by using the highest residual activity as 100%. Data represent the mean ± standard deviation of triplicate measurements.

The influence of reaction temperature on the catalytic activity of RsCgk was studied and the results are shown in Figure 3A. RsCgk showed maximum activity at the temperature of 40 °C. In the temperature range of 30–50 °C, RsCgk displayed over 60% of the highest activity detected at the optimal temperature. When the temperature exceeded 60 ℃, the relative enzyme activity of recombinant RsCgk was less than 32%. The optimal temperature of RsCgk was the same as other κ-carrageenases derived from *Pedobacter hainanensis* NJ-02 [16], *Pseudoalteromonas porphyrae* LL1 [25], *Pseudoalteromonas carrageenovora* ASY5 [12], and *Pseudoalteromonas carrageenovora* Psc [26]. 

The thermal stability of RsCgk was evaluated at the temperatures of 30 °C, 35 °C, and 40 °C. As shown in Figure 3B, RsCgk was relatively stable at 30 °C. After incubation at 30 ℃ for 120 min, RsCgk still retained more than 50% of its initial activity. The thermal stability of RsCgk was shown to be inferior to most κ-carrageenases. We speculated that this was due to its adaptation to the marine environment and its poor stability in low-salt environments. Similar salt stabilization was also seen in proteases from *Bacillus pseudofirmus* [27]. Its optimal temperature rose from 50 °C to 80 °C with 0–3 M NaCl addition.

The optimum reaction pH of RsCgk was studied by measuring enzyme activities in buffers of different pH (5.0–10.0). As shown in Figure 3C, RsCgk exhibited the highest activity at pH 7.0. RsCgk exhibited over 60% relative activity in solutions of relatively neutral pH (6.0–8.0). Only negligible activity was detected at pH 5.0 and 10.0, indicating RsCgk was sensitive to strong acidic and alkaline reaction pHs. A similar phenomenon was seen in the study of κ-carrageenase from *Pseudoalteromonas* sp. QY203 [28]. The pH stability of RsCgk was evaluated by assaying the residual activity after incubating RsCgk in buffers of different pH (5.0–10.0) for 24 h at 4 °C (Figure 3D). RsCgk showed the highest pH stability at pH 7.0 and retained more than 75% of the relative residual activity within a pH range of 6.0–9.0. A strong alkaline pH (10.0) deactivated RsCgk.

The Michaelis–Menten kinetic constant (*K*_m_) and *k*_cat_ for RsCgk were calculated using nonlinear fitting according to the Michaelis–Menten equation. The *K*_m_ and *k*_cat_ value of RsCgk for κ-carrageenan were 8.14 mg/mL and 151.4 s^−1^. The *K*_m_ value was lower than κ-carrageenase from *Pseudoalteromonas* sp. (9.8 mg/mL) [29]. Car1383 from the metagenome of antarctic macroalga-associated bacteria (*K*_m_ = 6.51 mg/mL) [30], CgkHC4 from *Tamlana* sp. HC4 (*K*_m_ = 7.63 mg/mL) [31], CgkS from *Shewanella* sp. Kz7 (*K*_m_ = 0.15 mg/mL) [32], and CgkZ from Zobellia sp. ZM-2 (*K*_m_ = 2.07 mg/mL) [33] exhibited lower *K*_m_ values than those in our study. 

### 2.4. Degradation Pattern of RsCgk

**Figure 4:**
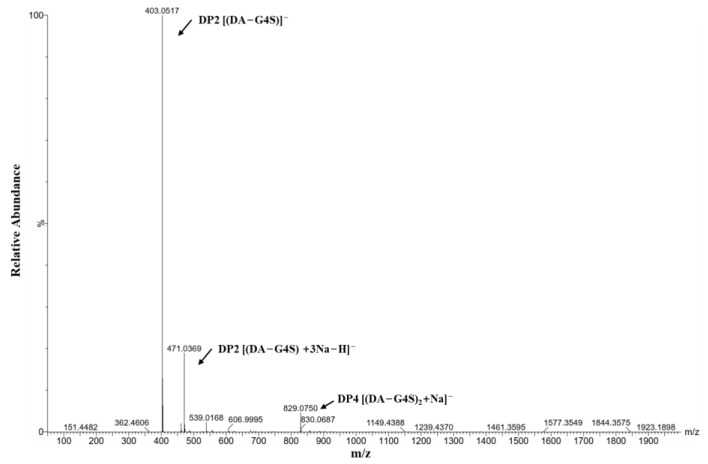
ESI-MS analysis of the hydrolysis products of RsCgk with κ-carrageenan as substrate.

After 24 h of degradation reaction, the RsCgk degradation product was analyzed using ESI-MS. As shown in Figure 4, the main degradation product of κ-carrageenan using RsCgk is κ-carrabiose. Only a small amount of κ-carratetraose was detected. There was no κ-carrahexaose or other κ-carrageenan oligosaccharides with higher degrees. Most reported κ-carrageenase [30,34] degraded κ-carrageenan into κ-carrageenase oligosaccharides with different forms (κ-carrabiose, κ-carratetraose, κ-carrahexaose, etc.), which was difficult for oligosaccharide purification and application. Our results indicated that RsCgk could effectively hydrolyze κ-carrageenan into uniform κ-carrabiose, which was beneficial for reducing the cost of further oligosaccharide purification and the production of high-value-added carrageenan products. Except for our study, there are currently only two κ-carrageenase from *Polaribacter* sp. NJDZ03 [8] and *Vibrio* sp. SY01 [9] that are able to specifically degrade κ-carrageenan into κ-carrabiose.

### 2.5. Salt Stabilization and Salt Tolerance of RsCgk

**Figure 5:**
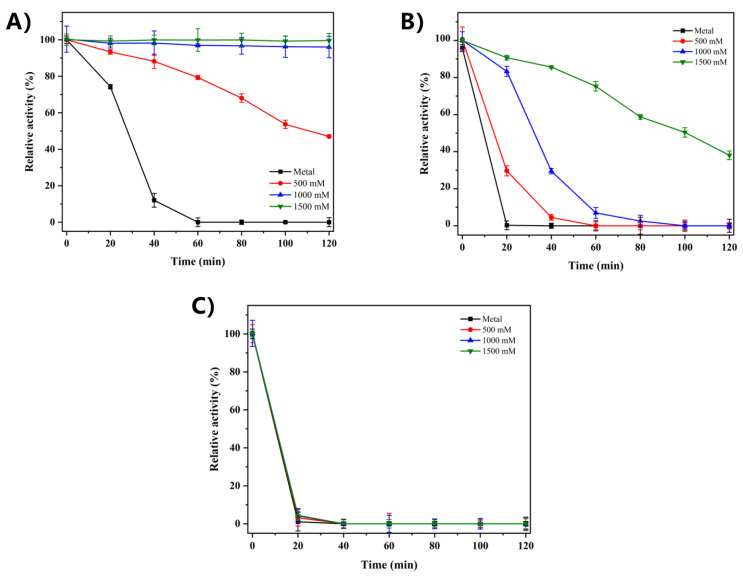
The effects of different NaCl concentrations (0–1500 mM) on the thermal stability of RsCgk. (**A**) 35 ℃. (**B**) 40 ℃. (**C**) 45 ℃. Residual RsCgk activities were calculated by using the initial residual activity as 100%. Data represent the mean ± standard deviation of triplicate measurements.

The thermal stability of recombinant RsCgk is insufficient for industrial applications at the relatively high temperatures required, and it is completely inactivated by 20 min incubation at 40 °C. Considering that marine-derived enzymes may require a salt environment to stabilize their conformations, the effect of salt concentration on the thermal stability of RsCgk was investigated at 35 °C, 40 °C, and 45 °C. As shown in Figure 5A, after 60 min incubation at 35 °C, the residual activities of RsCgk with 0 mM, 500 mM, 1000 mM, and 1500 mM NaCl were 0%, 79.30%, 96.94%, and 99.86%, respectively. When the temperature was increased to 40 °C, RsCgk with 0 mM, 500 mM, 1000 mM, and 1500 mM NaCl retained 0%, 4.52%, 29.48%, and 85.64% of its residual activity after a 60 min incubation (Figure 5B). The results showed that the presence of NaCl at 35 °C or 40 °C can greatly enhance the thermal stability of RsCgk. The enzyme activity could be fully maintained at 35 °C for 120 min with 1500 mM NaCl. Moreover, the thermal stability of RsCgk was significantly increased by a high salt concentration (1500 mM) at 40 °C compared to at 35 °C. The aforementioned results suggest that RsCgk is a typical marine-derived enzyme that depends on a salt environment to maintain its structural stability. However, the thermal stability of RsCgk at 45 °C did not exhibit an increase with the addition of NaCl (Figure 5C), indicating that salt addition was not sufficient to overcome its sensitivity to higher temperatures.

**Figure 6:**
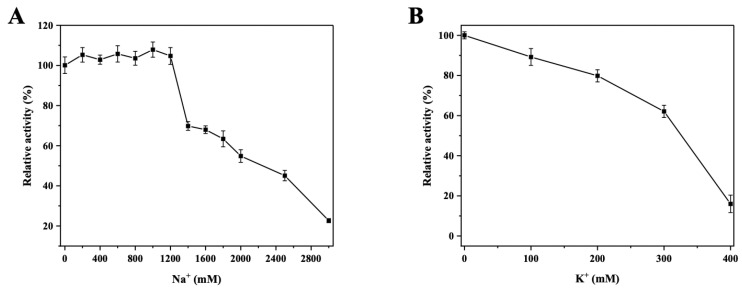
Effects of (**A**) NaCl (0–3000 mM) and (**B**) KCl (0–400 mM) concentration on the enzymatic activity of RsCgk. Relative RsCgk activities were calculated using the activity obtained without salts as 100%. Data represent the mean ± standard deviation of triplicate measurements.

The extraction of carrageenan usually includes an alkali treatment (NaOH [18] or KOH [35]) of the raw red algae, which results in a high concentration of sodium and potassium salts remaining in the carrageenan extraction wastes, impeding the further utilization of these wastes. Therefore, κ-carragenases with high salt tolerances are needed for reuse of the carrageenan extraction wastes to achieve efficient, environmentally friendly processes. As shown in Figure 6A, the enzyme activity of RsCgk was maintained between 102.88–107.89% in 0–1200 mM NaCl. Compared with other κ-carrageenases, RsCgk exhibited adequate NaCl tolerance. κ-carragenase from *Wenyingzhuangia fucanilytica* CZ1127 [34] was able to maintain approximately 50% of its activity with 500 mM NaCl. With the addition of 1000 mM NaCl, the activity of κ-carragenase from *Wenyingzhuangia aestuarii* OF219 [36] was completely lost, while κ-carragenase from *Pedobacter hainanensis* NJ-02 [16] retained about 32% of its activity. Xiao et al. reported that κ-carragenase from *Pseudoalteromonas carrageenovora* ASY5 [17] retained more than 50% of its highest activity at 1800 mM NaCl, which was the highest reported NaCl tolerance of κ-carragenase. However, the activities of this κ-carragenase at lower NaCl concentrations were particularly low (less than 10% at 200 mM NaCl and less than 20% at 600 mM NaCl), which may cause problems in practical applications. In addition, unlike many κ-carragenases, RsCgk showed full activity without NaCl, which would be beneficial for the production of carrageenan oligosaccharides.

Potassium ions show great differences in the enzymatic activity of κ-carrageenases of different sources. The activity of κ-carrageenase from *Tamlana* sp. HC4 [31] was critically inhibited by 50.87% with only 1 mM K^+^, while 100 mM K^+^ reduced the activity of κ-carrageenase from the *Cellulophaga lytica strain* N5-2 [15] by 76.40%. κ-carrageenase from *Pseudoalteromonas carrageenovora* ASY5 [17] exhibited enhanced activity (160%) with the addition of 100 mM K^+^, but completely lost activity at 200 mM K^+^. Our study found that RsCgk could retain 89.23%, 79.84%, 62.11%, and 16.01% of its activity in 100 mM, 200 mM, 300 mM, and 400 mM KCl (Figure 6B), which exhibited the highest potassium salt tolerance among current reports. The ability to perform a degradation reaction at a high concentration of sodium and potassium ions makes RsCgk a suitable tool for utilizing carrageenan extraction wastes.

### 2.6. Structural Insight into the Potassium Tolerance of RsCgk

**Figure 7:**
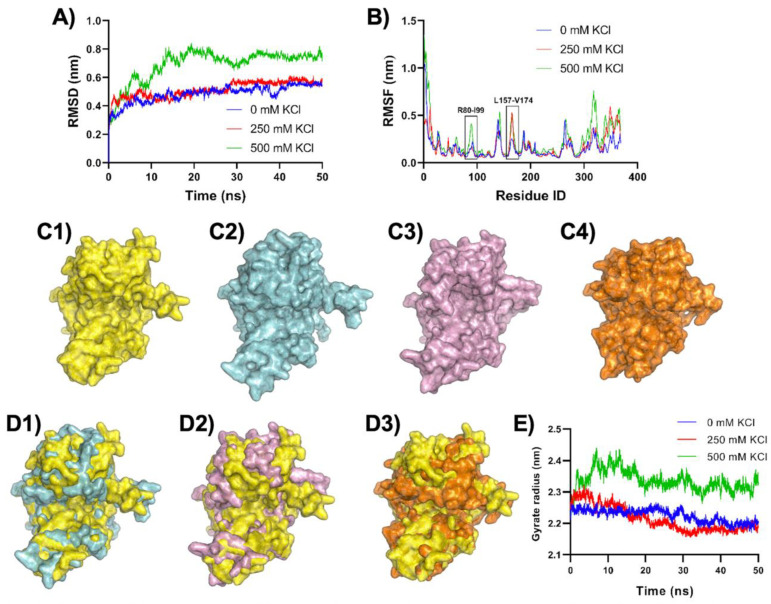
(**A**) RMSDs of RsCgk in 0 mM, 250 mM, and 500 mM KCl during 50 ns MD simulations as a function of time relative to its initial structure. (**B**) RMSFs of RsCgk in 50 ns MD simulations of RsCgk in 0 mM, 250 mM, and 500 mM KCl. The most affected domains, Loop R80-I99 and Loop L157-V174, are highlighted using black boxes. (**C1**) The surface presentation of RsCgk with salt addition. (**C2**) The surface presentation of RsCgk with 250 mM KCl. (**C3**) The surface presentation of RsCgk with 500 mM KCl. (**C4**) The surface presentation of RsCgk with 500 mM NaCl. (**D1**) The surface comparison of RsCgk with and without 250 mM KCl. (**D2**) The surface comparison of RsCgk with and without 500 mM KCl. (**D3**) The surface comparison of RsCgk with and without 500 mM NaCl. (**E**) Gyrate radius of RsCgk in 0 mM, 250 mM, and 500 mM KCl during 50 ns MD simulations.

MD simulations were performed to gain insight into the effect of potassium ions on the activity of RsCgk. MD simulation of RsCgk in 0 mM, 250 mM, and 500 mM KCl was performed at 30 °C for 50 ns. RMSDs in 50 ns MD simulations were analyzed using the initial structure of RsCgk as a reference (Figure 7A). The average RMSDs of RsCgk at 0 M, 250 mM, and 500 mM KCl salt concentrations were 0.485 nm, 0.514 nm, and 0.689 nm, respectively. The addition of 250 mM KCl did not affect RMSD of RsCgk much, which was in accordance with the experimental result that a low concentration of KCl had relatively weak inhibition of RsCgk activity. On the contrary, the difference in RMSD between the 0 M and 500 mM KCl was obviously larger, with an average RMSD increase of 42.06%. We speculate that excessive structural perturbation may be responsible for the reduction in RsCgk activity. MD trajectories for RsCgk were depicted in terms of RMSFs to further elucidate potential salt-affected structural variations. Figure 7B shows that the domain most affected by KCl was Loop R80-I99 and Loop L157-V174. The average RMSF of Loop R80-I99 in 500 mM KCl was 0.200 nm, which was enhanced by 70.85% compared with that without KCl (0.117 nm). The average RMSF of Loop L157-V174 in 500 mM KCl (0.261 nm) was enhanced by 66.96% compared with that without KCl (0.156 nm). These loops were critical for the formation of the catalytic tunnel that was related to κ-carrageenan binding and oligosaccharide release. 

The drastic influence of the KCl addition on the tunnel-forming loops was further investigated by analysis of RsCgk conformation changes due to KCl interaction. The average conformations of RsCgk with 0 mM, 250 mM, and 500 mM KCl within 50 ns simulation were obtained and are shown in Figure 7C1–C3. It was obvious that the tunnel-forming loops (Loop R80-I99, Loop L157-V174, and L256-E283) of RsCgk in 500 mM KCl turned outward toward active tunnels, which may have had a negative effect on the formation of catalytic tunnels. When 250 mM KCl was added, only Loop L157-V174 had a relatively slight turning outward phenomenon compared with that in 500 mM KCl. The protein surface comparisons are also shown in Figure 7D1,D2. The catalytic tunnel of RsCgk was clearly expanded with the addition of a high concentration of KCl. The structural expansion of RsCgk in a high concentration of KCl could be also demonstrated by the change in the radius of gyration (Figure 7E). The average radius of gyration of RsCgk with 0 mM, 250 mM, and 500 mM KCl within 50 ns simulation were 2.22 nm, 2.21 nm, and 2.33 nm. The radius of gyration of RsCgk with 500 mM KCl was increased by 0.11 nm compared with that without KCl.

**Figure 8:**
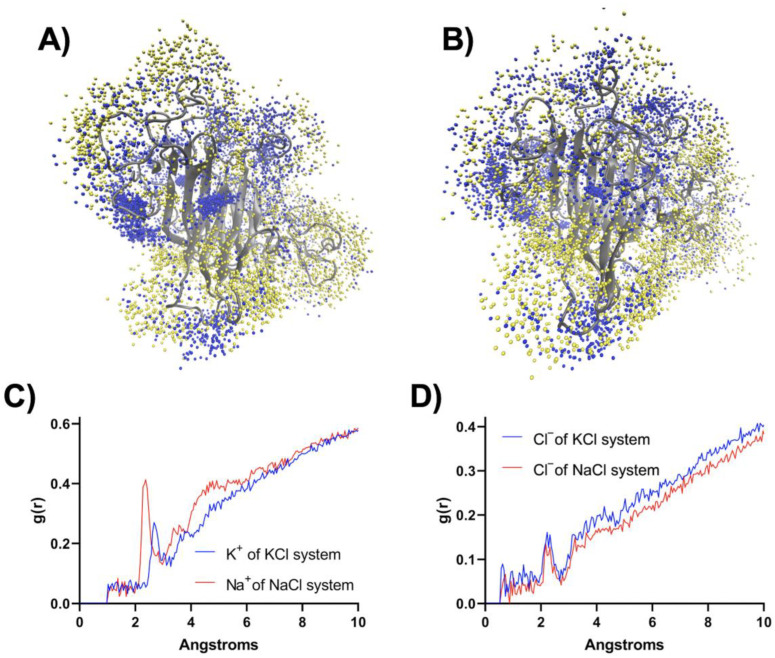
(**A**) The distribution of K^+^ (depicted as blue spheres) and Cl^−^ (depicted as yellow spheres) within 3 Å of the RsCgk surface. The presentation includes K^+^ and Cl^−^ from 200 frames covering the 50 ns MD trajectory of RsCgk in 500 mM KCl. (**B**) The distribution of Na^+^ (depicted as blue spheres) and Cl^−^ (depicted as yellow spheres) within 3 Å of the RsCgk surface. The presentation included Na^+^ and Cl^−^ from 200 frames covering the 50 ns MD trajectory of RsCgk in 500 mM NaCl. (**C**) Radial distribution functions (g(r)) for K^+^ and Na^+^ against RsCgk in 500 mM KCl or NaCl. RDFs were calculated using 200 frames of the 50 ns MD trajectory. (**D**) Radial distribution functions (g(r)) for Cl^−^ against RsCgk in 500 mM KCl or NaCl. RDFs were calculated using 200 frames of the 50 ns MD trajectory.

Moreover, we implemented MD simulation for RsCgk in 500 mM NaCl to compare the effect of different cations (K^+^ or Na^+^) on the RsCgk structure. It can be seen from Figure 7C4,D3 that when 500 mM NaCl was added, the tunnel-forming loops of RsCgk did not appear to turn outward as in the case of 500 mM KCl, but appeared to turn inward to form a more compact tunnel. The dynamic pattern of the tunnel-forming loops of RsCgk was related to the interaction between ions and enzymes. As shown in Figure 8A,B, the population of K^+^ cations associated with RsCgk was significantly lower than that of Na+ cations at the same concentration, especially near the tunnel-forming loops (Loop R80-I99, Loop L157-V174, and L256-E283), and this also caused more Cl^−^ anions to occupy the respective area. Radial distribution functions (RDFs) were also analyzed to interpret the interaction of K^+^, Na^+^, and Cl^−^ with RsCgk surfaces (Figure 8C,D). The results of RDFs showed that the density of Na^+^ cations near RsCgk was higher than that of K^+^ cations, especially within 6 Å, indicating that Na^+^ had a stronger interaction with RsCgk. Cl^−^ anions in the RsCgk-KCl system were also displayed at higher density than in the RsCgk-NaCl system. The shortest association peaks for K^+^ and Na^+^ occurred at 2.375 Å and 2.525 Å, whereas that for Cl^−^ was at about 2.30 Å. Na^+^ showed a shorter association distance with RsCgk, indicating a better coordination ability than K^+^, which may explain the cation density difference. These results indicated that the conformational difference of these tunnel-forming loops under different salt environments may lead to deactivation or stabilization of RsCgk. The loops near the active site were reported to be critical for other seaweed depredating enzymes such as alginate lyase [37,38,39]. Future structural analysis and experiments should be performed to achieve full understanding of the salt–potassium tolerance of κ-carragenase.

## 3. Materials and Methods

### 3.1. Materials

κ-carrageenans, ι- carrageenans, and λ- carrageenans were purchased from Yuanye Bio-Technology (Shanghai yuanye Bio-Technology Co., Ltd., Shanghai, China). Other chemicals used in this study were of analytical grade.

### 3.2. Construction of Recombinant E. coli for Expressing RsCgk

A κ-carrageenase gene (GenBank ID: EMI53032.1) from marine bacterium Rhodopirellula sallentina SM41 was selected through database mining. The complete gene was codon-optimized and then synthesized by Sangon Biotech (Shanghai, China), inserted into a pET28a(+) plasmid using NdeI and XhoI as restriction digestion sites, and then transformed into E. coli BL21 (DE3) competent cells for recombinant protein expression.

### 3.3. Expression and Purification of RsCgk

The recombinant E. coli containing the RsCgk gene was grown in LB medium containing 50 μg/mL kanamycin at 37 °C with horizontal shaking at 180 rpm. A total of 0.5 mM IPTG was added to induce the expression of RsCgk when OD_600_ reached 0.6–0.8. The recombinant E. coli was then cultivated at 16 °C with horizontal shaking at 180 rpm. After another 20 h incubation, the E. coli cells were harvested by centrifugation, and ultra-sonicated and centrifuged at 8000 rpm for 20 min at 4 °C to attain crude enzyme. The recombinant RsCgk protein was purified using an Ni-NTA (GE Healthcare, Chicago, IL, USA) column. Eluted fractions with Rscgk activity were combined and replaced with Na_2_HPO_4_-NaH_2_PO_4_ buffer (50 mM, pH 7.0) using a Macrosep Advance centrifugal device (Pall, East Hills, NY, USA; cut-off distance, 5 kDa). The enzyme purity was tested using SDS-PAGE.

### 3.4. Measurement of RsCgk Activity

For the determination of RsCgk activity, the 3,5-dini-trosalicylic acid (DNS) method was applied. A total of 100 μL of the purified recombinant RsCgk was mixed with 500 μL of κ-carrageenan (0.2%, dissolved in 50 mM Na_2_HPO_4_-NaH_2_PO_4_ buffer, pH 7.0) and allowed to react at 40 °C for 30 min. Then, 900 μL of DNS solution was added into the reaction mixture, boiled for 10 min for the chromogenic reaction, and then the absorbance at 520 nm was assayed. The standard curve for reducing sugar was obtained using D-galactose. One unit of RsCgk activity was defined as the amount of enzyme required to catalyze the production of 1 μmol reducing sugar (equivalent to D-galactose) per minute.

### 3.5. Effects of Temperature and pH on RsCgk Activity and Stability

RsCgk was measured for its activity with the variation of temperature (10–70 °C) to investigate the effect of temperature on RsCgk activity. Relative RsCgk activities were calculated using the activity obtained at the optimum temperature as 100%. For the evaluation of thermal stability, RsCgk was kept at different temperatures (30 °C, 35 °C, 40 °C) for 120 min to investigate the effect of temperature on RsCgk thermal stability. Residual RsCgk activities were calculated by using the initial activity as 100%.

RsCgk activities were determined under the assay conditions described previously to study the effect of pH on RsCgk activity. The buffers used for the test included Na_2_HPO_4_–citric acid buffer (50 mM, pH 5.0–6.0), Na_2_HPO_4_–NaH_2_PO_4_ buffer (50 mM, pH 6.0–8.0), Tris–HCl buffer (50 mM, pH 8.0–9.0), and glycine–sodium hydroxide buffer (50 mM, pH 9.0–10.0). Relative RsCgk activities were calculated using the activity measured at optimum pH as 100%. RsCgk was incubated in the buffers described above (50 mM, pH 5.0–10.0) at 4 °C for 24 h to study the effect of pH on RsCgk stability. Residual RsCgk activities were calculated by using the highest residual activity as 100%.

### 3.6. Kinetic Parameters of RsCgk

The kinetic parameters of the purified RsCgk were studied by assaying the enzyme activity in different κ-carrageenan concentrations (1–10 mg/mL). The kinetic parameter was then calculated using nonlinear fitting according to the Michaelis–Menten equation. 

### 3.7. ESI-MS Analysis

A total of 500 µL of RsCgk was added into 500 µL of the substrate solution (0.5% κ-carrageenan) to study the degradation pattern of RsCgk. The degradation reaction was allowed to proceed at 30 °C for 24 h. The reaction samples were withdrawn periodically and tested for the reducing sugar concentration. When the reducing sugar concentration was stable, the reaction mixture was boiled for 10 min to inactivate the RsCgk and centrifuged at 10,000× *g* for 15 min at 4 °C. The supernatant was withdrawn and added to absolute ethanol (3-fold volume of reaction mixture). The mixture was incubated at 4 °C for 8 h and then centrifuged at 10,000× *g* for 20 min at 4 °C. The supernatant was withdrawn and concentrated by rotary evaporation and then lyophilized.

The degradation product of RsCgk was determined using electrospray ionization mass spectrometry (ESI-MS) (Bruker Esquire HCT, Billerica, MA, USA). The lyophilized sample was dissolved in chromatographic grade methanol with a concentration of 10 mg/mL. A total of 2 µL of the degradation products was loop-injected into the ESI-MS instrument operated in negative-ion mode with the following settings: calibration dynamics, 2; capillary voltage, 4.00 kV; cone voltage, 20.00 V; source temperature, 150 °C; desolvation temperature, 350 °C; cone gas flow rate, 50 L/h; and desolvation gas flow, 500 L/h.

### 3.8. Effects of Salts on RsCgk

The effects of salts on RsCgk activity were studied under standard assay conditions with different concentrations of NaCl (0–3000 mM) or KCl (0–400 mM). Relative RsCgk activities were calculated using the activity obtained without salts as 100%. 

The effects of salts on RsCgk thermal stability were evaluated under standard assay conditions with different concentrations of NaCl (0–1500 mM). Residual RsCgk activities were calculated by using the initial residual activity as 100%.

### 3.9. MD Simulation

The protein structure of RsCgk was constructed using the I-TASSER server (http://zhanglab.ccmb.med.umich.edu/I-TASSER, accessed on 8 August 2022). MD simulations were implemented using the Gromacs (version: 2019.6 release) and the AMBER 99SB force field. Solvent molecule modeling was performed using the TIP3P water model. K^+^ (Na^+^) and Cl^−^ were used for the neutralization of the simulation system. Minimizations were executed with the steepest descent integrator until maximum force was below 1000 kJ mol^−1^ nm^−1^ on each atom. The truncation radius for Van der Waals interactions was 10 Å, while electrostatic interactions were truncated at 9 Å using a twin-range cutoff method. The particle mesh Ewald method was used to treat the long-range electrostatic interactions. The bonds linked with hydrogen atoms were restrained with a linear constraint solver. The pressure during simulation was fixed at 1.0 atm using a Parrinello–Rahman barostat with a coupling constant of 2 ps. Simulations were performed with a 2 fs timestep. All MD simulations were firstly equilibrated under a constant-volume (NVT) ensemble for 100 ps and then under a constant-pressure (NPT) ensemble for 100 ps, and the MD simulation procedure was carried on for 50 ns. RMSD, RMSF, and the radius of gyration were calculated using the standard tools of the GROMACS package. Radial distribution functions (g(r)) were calculated with VMD 1.9.3. Protein structure images were prepared using PyMOL (version:2.5, Schrodinger, LLC, New York, NY, USA).

## 4. Conclusions

In summary, a novel κ-carrageenase RsCgk from the marine bacterium *Rhodopirellula sallentina* SM41 was identified and heterologously overexpressed in *Escherichia coli* BL21. The similarity of RsCgk with κ-carrageenases from different genus was less than 65%. The optimal temperature and pH of RsCgk were 40 °C and 7.0, respectively. RsCgk could effectively hydrolyze κ-carrageenan into uniform κ-carrabiose, which was beneficial for further application in oligosaccharides production. The thermal stability of RsCgk was greatly enhanced with the addition of NaCl. Compared with other κ-carrageenases, RsCgk exhibited great salt tolerant properties, especially for KCl, which could support the utilization of carrageenan waste and achieving environmentally friendly extraction processes. A conformational structural feature was observed in MD simulation analysis to explain the salt-deactivation phenomenon of RsCgk. Comparative analysis and loop engineering will be implemented for further elucidation of the salt-regulation properties of κ-carrageenase.

## Data Availability

The data that support the findings of this study are available from the corresponding authors upon reasonable request.
